# The influences of cognitive appraisal, physical injury, coping strategy, and forgiveness of others on PTSD symptoms in traffic accidents using hierarchical linear modeling: Erratum

**DOI:** 10.1097/MD.0000000000009130

**Published:** 2017-12-01

In the article, “The influences of cognitive appraisal, physical injury, coping strategy, and forgiveness of others on PTSD symptoms in traffic accidents using hierarchical linear modeling”,^[1]^ that appeared in Volume 96, Issue 35 of *Medicine*, the authors would like to add that the article is partly extracted and modified from Dr. Bae's PhD thesis.
